# Heterodimeric IL-15 (hetIL-15) reduces circulating tumor cells and metastasis formation improving chemotherapy and surgery in 4T1 mouse model of TNBC

**DOI:** 10.3389/fimmu.2022.1014802

**Published:** 2023-01-13

**Authors:** Vasiliki Stravokefalou, Dimitris Stellas, Sevasti Karaliota, Bethany A. Nagy, Antonio Valentin, Cristina Bergamaschi, Konstantinos Dimas, George N. Pavlakis

**Affiliations:** ^1^ Human Retrovirus Section, Vaccine Branch, Center for Cancer Research, National Cancer Institute at Frederick, Frederick, MD, United States; ^2^ Department of Pharmacology, Faculty of Medicine, University of Thessaly, Larissa, Greece; ^3^ Department of Chemical Biology, National Hellenic Research Foundation, Athens, Greece; ^4^ Basic Science Program, Frederick National Laboratory for Cancer Research, Leidos Biomedical Research, Frederick, MD, United States; ^5^ Laboratory Animal Sciences Program, Frederick National Laboratory for Cancer Research, Leidos Biomedical Research, Frederick, MD, United States; ^6^ Human Retrovirus Pathogenesis Section, Vaccine Branch, Center for Cancer Research, National Cancer Institute at Frederick, Frederick, MD, United States

**Keywords:** heterodimeric IL-15 (hetIL-15), doxorubicin, chemoimmunotherapy, circulating tumor cells (CTCs), metastasis, surgery, triple negative breast cancer (TNBC)

## Abstract

Immunotherapy has emerged as a viable approach in cancer therapy, with cytokines being of great interest. Interleukin IL-15 (IL-15), a cytokine that supports cytotoxic immune cells, has been successfully tested as an anti-cancer and anti-metastatic agent, but combinations with conventional chemotherapy and surgery protocols have not been extensively studied. We have produced heterodimeric IL-15 (hetIL-15), which has shown anti-tumor efficacy in several murine cancer models and is being evaluated in clinical trials for metastatic cancers. In this study, we examined the therapeutic effects of hetIL-15 in combination with chemotherapy and surgery in the 4T1 mouse model of metastatic triple negative breast cancer (TNBC). hetIL-15 monotherapy exhibited potent anti-metastatic effects by diminishing the number of circulating tumor cells (CTCs) and by controlling tumor cells colonization of the lungs. hetIL-15 treatment in combination with doxorubicin resulted in enhanced anti-metastatic activity and extended animal survival. Systemic immune phenotype analysis showed that the chemoimmunotherapeutic regimen shifted the tumor-induced imbalance of polymorphonuclear myeloid-derived suppressor cells (PMN-MDSCs) in favor of cytotoxic effector cells, by simultaneously decreasing PMN-MDSCs and increasing the frequency and activation of effector (CD8^+^T and NK) cells. Tumor resection supported by neoadjuvant and adjuvant administration of hetIL-15, either alone or in combination with doxorubicin, resulted in the cure of approximately half of the treated animals and the development of anti-4T1 tumor immunity. Our findings demonstrate a significant anti-metastatic potential of hetIL-15 in combination with chemotherapy and surgery and suggest exploring the use of this regimen for the treatment of TNBC.

## Introduction

Breast cancer is the leading cause of cancer-related deaths in women globally, according to CLOBOCAN (1). One of the most challenging breast cancer subtypes is the triple-negative breast cancer (TNBC), which is characterized by the lack of estrogen receptor, progesterone receptor and human epidermal growth factor receptor 2 (HER2) expression (2). TNBC accounts for 10% to 20% of all breast carcinomas and has a poor prognosis (3). TNBC is more likely to show metastatic recurrence and patients with metastatic TNBC have higher mortality compared to patients with other breast cancer types (42.2% vs 28%, respectively) (4). This is attributed mostly to the lack of therapeutic targets and to the acquired resistance of tumor cells from previous chemotherapeutic treatments (5, 6), which support the need for additional therapeutic strategies.

The recent introduction of immunotherapeutic interventions has resulted in favorable outcomes in several human cancers (7) and provides additional opportunities for new therapeutic combinations. IL-15 is a cytokine of great interest to the cancer immunotherapy field (8); it enhances anti-tumor responses through the stimulation of several leukocyte populations, including cytotoxic CD8^+^ T lymphocytes and NK (Natural Killer) cells (9–11). Early studies showed that recombinant single chain IL-15 (rhIL-15) produced in *Escherichia coli* has substantial anti-tumor activity (12). Additional efforts to improve IL-15 *in vivo* properties led to the generation of several more stable variants that combine IL-15 with IL-15Rα. The promising results of these variants in several preclinical cancer models have advanced them to clinical trials for safety and efficacy evaluation [reviewed in (13, 14)]. Studies by our group revealed that IL-15 is produced together with IL-15Rα, forming a heterodimeric protein that is the *in vivo* active form of the cytokine in mice and humans (15–18). IL-15Rα is therefore part of the cytokine and does not have any receptor function (13). The complex is termed heterodimeric IL-15 (hetIL-15) and has an extended half-life and better efficacy compared to rhIL-15 (15, 17). Our studies have shown that hetIL-15 exhibits significant anti-cancer activity as monotherapy in several preclinical cancer models including B16 melanoma, MC38 colon carcinoma, EO771 breast adenocarcinoma and TC-1 carcinoma (19–21). Additionally, we have reported that hetIL-15 enhances adoptive cell transfer (ACT) in immunocompetent hosts promoting T lymphocyte infiltration into the tumor (19). hetIL-15 (NIZ985) has been tested as a single agent in a first-in-human study (22), and is currently being evaluated in combination with anti-PD-1 antibodies (Spartalizumab and Tislelizumab) for the treatment of metastatic or unresectable tumors (NCT02452268) and solid tumors or lymphoma (NCT04261439). Results from the phase I NCT02452268 clinical trial showed that hetIL-15 as a single agent is well tolerated and induces IFN-γ production and expansion of cytotoxic lymphocytes in patients with several advanced cancers (22).

Several preclinical studies have demonstrated the important role of IL-15 in controlling metastatic disease (23–29), and combinations with immune checkpoint inhibitors (ICIs) have been proven beneficial (27, 30). In spite of initial supportive evidence (31), IL-15 anti-metastatic activity has not been extensively tested in combination with chemotherapeutic agents. It has been shown that low-dose chemotherapy can enhance anti-tumor immune responses by inducing immunogenic cell death (32), providing the rationale for combining chemotherapy with immunotherapy. To explore novel and more effective therapeutic interventions against metastatic TNBC, we studied the anti-cancer effects of hetIL-15 in combination with the chemotherapeutic agent doxorubicin in the 4T1 murine model of TNBC. Doxorubicin is a broadly used chemotherapeutic agent of the anthracyclines class and the foremost standard of care for TNBC (33). In the present study, we examined the anti-metastatic effects of the treatment by monitoring the presence of metastatic tumor cells in both blood and lungs. To study the mechanism of action of this chemo-immunotherapeutic regimen, we further analyzed the immune cell landscape in the blood, spleen, lung and tumor focusing on the cytotoxic effector cells (CD8^+^T and NK cells) and immunosuppressive populations (MDSCs). Finally, following standard protocols applicable in clinical settings, we evaluated the efficacy of hetIL-15 alone or together with doxorubicin as neoadjuvants and adjuvants in combination with surgery. Our study evaluates for the first time the therapeutic effect of hetIL-15 in combination with doxorubicin, with or without surgery, demonstrating a significant anti-metastatic potential of this regimen.

## Materials and methods

### Cells and mice

4T1 cell line (ATCC) was tested for mycoplasma (by PCR using standard mycoplasma testing protocol) and cultured in RPMI-1640 [+] L-glutamine medium (Gibco, #11875-093) supplemented with 100 IU/ml penicillin and 100 μg/ml streptomycin (Lonza, # DE17-602E) and 10% heat-inactivated fetal bovine serum (FBS, Sigma, #F2442). Female Balb/c mice were purchased from Charles River Laboratories (Wilmington, MA, USA) and kept under pathogen-free conditions at the National Cancer Institute Animal Facility in Frederick.

The studies were approved by the National Cancer Institute-Frederick Animal Care and Use Committee. NCI-Frederick is accredited by AAALAC International and follows the Public Health Service Policy for the Care and Use of Laboratory Animals. Animal care was provided in accordance with the procedures outlined in the “Guide for Care and Use of Laboratory Animals (National Research Council; 1996; National Academy Press; Washington, D.C.).

### Animal studies and treatment

4T1 tumor cells (0.35-1x10^6^) were orthotopically inoculated in the fourth mammary fat pad or injected (10^4^) in the lateral tail-vein (intravenously, IV) of 6-8 weeks old Balb/c mice. For the orthotopic inoculation, the cells were resuspended in Dulbecco’s PBS (DPBS, Gibco, # 14190-144), and Matrigel (Corning, #354234) was added at 1:3 dilution. Matrigel is a soluble and sterile extract of basement membrane proteins that forms a 3D gel at 37°C (34) preventing cell leakage to adjacent tissues upon inoculation. Tumor size was measured by a digital caliper and tumor volume was calculated by the equation L*W*H*π/6. The treatment started when the tumors reached a size of 30-80mm^3^. In both orthotopic and IV models, the mice were randomized into four therapeutic groups: untreated (PBS) (eight IP injections), doxorubicin [(Doxorubicin Hydrochloride injection solution (Dox), Pfizer, #NDC 0069-4037)] (three IV injections - 5mg/kg), hetIL-15 (eight IP injections - 3ug/mouse) and doxorubicin+hetIL-15 (combined schedule of monotherapies). hetIL-15 was purified from HEK293 cells (Admune Therapeutic LLC/Novartis). The study endpoints were: (i) day 22 and (ii) day 18, post tumor cell inoculation, in the orthotopic and IV model, respectively. In the survival studies, mice were sacrificed when the primary tumor reached a 2cm diameter or any other humane endpoints listed in the ACUC-approved animal protocol, such as 20% weight loss or acute morbidity.

### Metastasis evaluation

Metastasis evaluation in the blood and lungs was performed by clonogenic assays according to Pulaski and Ostrand-Rosenberg protocol (35). Briefly, RBC-lysed blood and lung cell suspensions were placed in a petri dish (100 mm) and cultured in a selection medium: complete RPMI-1640 [+] L-glutamine supplemented with 60μg/ml of 6-thioguanine (6-TG, Sigma, #A4882) for 14 days. Lung cell suspensions were obtained by enzymatic digestion (Lung Dissociation Kit, mouse, Miltenyi Biotec, #130-095-927) and mechanical dissociation (GentleMACS dissociator, Miltenyi Biotec) according to the manufacturer’s protocol. At the end of the 14 days, colonies were fixed with 50% ice-cold trichloroacetic acid (TCA, Sigma, #T6399) and stained with sulforhodamine B (0.04% SRB, Sigma, #S1402) according to Orellana and Kasinski protocol (36). The evaluation was performed macroscopically under the stereoscope counting the individual colonies and assigning the animals in one of the following ranges: 0, 1-50, 50-500 and >500 number of colonies. For the India Ink staining, India Ink (15%, Speedball, #3378) was injected into the trachea of the animal and the lungs were washed in PBS. Lungs were then stored overnight in Fekete’s solution containing 300ml of 70% ethanol (Pharmco, #111000200), 30ml of 37% formaldehyde (Sigma, #252549) and 5ml of glacial acetic acid (Sigma, #A6283). The next day, the white pulmonary tumor nodules were counted macroscopically.

### Histological analysis

Lungs were fixed in 10% neutral buffered formalin (NBF, Sigma, #HT501128) and paraffin embedded. Sections were stained with hematoxylin/eosin (H&E) or processed for immunohistochemistry (IHC). IHC automated staining was performed on Leica Biosystems’ Bond RX with the following conditions: Epitope Retrieval 1 (Citrate) 20’ for CD8a (eBioscience, #14-0808-82, 1:50) and Ly6G/GR1 Granulocyte Marker (Origene, #DM3589P, 1:100). The Bond Polymer Refine Detection Kit (Leica Biosystems, #DS9800) with the omission of the Post Primary Reagent was used, and an anti-rat secondary antibody (Vector Labs, #BA-4001) was included. Isotype rat IgG2a antibody (BD Bioscience, #559073) was used in place of the primary antibodies for the negative controls. H&E and IHC slides were scanned using an Aperio AT2 scanner (Leica Biosystems, Buffalo Grove, IL) into whole slide digital images (one section was used for the analysis). Image analysis of positive-stained cells in lung tissue was performed using HALO image analysis software (v3.3.2541.300; Indica Labs, Corrales, NM). Positive-stained cells located in vessels or areas of artifact such as folds and tears were excluded from the analysis.

### Flow cytometry

Tumors and lungs were processed by enzymatic digestion and mechanical dissociation as mentioned above, and spleens were homogenized mechanically. Single-cell suspensions were obtained by filtering the homogenates using 100 μm cell strainers (Corning, #352360). Red blood cells were lysed using ACK lysis buffer (Lonza, #10-548E). Single cells were washed with PBS and stained (except blood) with a fixable aqua dead cell stain kit (ThermoFisher Scientific, #L34957) for 30 min at 4°C. Next, the samples were surface stained with the following antibodies: CD45 (clone 30-F11, Biolegend, #103108 or BD Biosciences, #557659), CD3 (clone 145-2C11, Biolegend, #100310), CD8a (clone 53-6.7, BD Biosciences, #563234), CD69 (clone H1.2F3, Biolegend, #104545), KLRG1 (clone 2F1, BD Biosciences, #563595), PD-1 (clone 29F.1A12, Biolegend, #135220), B220 (clone RA3-6B2, BD Biosciences, #553091), Ly6G (clone 1A8, BD Biosciences, #562700), CD49b (clone DX5, BD Biosciences, #562453) and Ly6C (clone AL-21, BD Biosciences, #560593), CD19 (clone 6D5, Biolegend, #115540 or clone 1D3, ThermoFisher Scientific, #15-0193-83), CD11b (clone M1/70, ThermoFisher Scientific, #45-0112-82). For intracellular staining, cells were fixed and permeabilized using the Foxp3 staining buffer (ThermoFisher Scientific, #00-5523-00), following the manufacturer’s instructions. After permeabilization, the cells were stained with Ki67 (clone B56, BD Biosciences, # 561277), granzyme B (clone GB12, ThermoFisher Scientific, #MHGB05) and bcl-2 (clone BCL/10C4, Biolegend, #633508). Samples were acquired in a Fortessa flow cytometer (BD Biosciences), and the data were analyzed using FlowJo software (Version 10.8.0, Becton Dickinson and Company, Ashland, OR). PMN-MDSCs were characterized as CD45^+^lin^neg^(CD3/CD49b/CD19/B220)/CD11b^+^/Ly6G^+^/Ly6C^low^ and M-MDSCs as CD45^+^lin^neg^(CD3/CD49b/CD19/B220)/CD11b^+^/Ly6G^−^/Ly6C^high^. Some of the tumors were excluded from the report of intratumoral cell populations because of an unusually high percentage of B cells (> 4.5% of CD45^+^) that was attributed to contamination with cells from the draining lymph node. All the animals were included in the tumor growth and survival analyses.

### Tumor surgeries and pre- and post-treatment

Tumor resections were performed one week post 4T1 cells (0.35 x 10^6^) inoculation (tumor volume 130mm^3^). The treatments started before and continued after surgery following a neoadjuvant and adjuvant setting. On day 4 (tumor size 60mm^3^), the mice were randomized into five groups: (1) untreated (PBS) without tumor resection, (2) untreated (PBS) with resection only, (3) doxorubicin-treated with resection, (4) hetIL-15-treated with resection (5) combination-treated with resection. The dosing schedule was the same as mentioned above except for the additional hetIL-15 administration on day 4 (total of nine injections). The study endpoint was either on day 22, or the mice were evaluated for their survival. Mice showing local tumor regrowth after surgery were excluded from the analysis.

### Rechallenge experiment

4T1 tumor cells (5x10^4^) were subcutaneously injected in the flanks of 18-20 weeks old naïve Balb/c mice (not previously challenged with 4T1 cells, control group) or survivors after tumor resection and hetIL-15 or combination therapy (considered as one group). Cells were resuspended in PBS and Matrigel was added at 1:3 dilution. The rechallenge was performed 90 days after the first challenge. The animals were monitored, and the tumors were measured for 19 days post-inoculation.

### Statistical analysis

Statistical analyses were performed using Prism 9.2.0 (GraphPad) Software (San Diego, CA, USA). Ordinary one-way ANOVA (analysis of variance) and Tukey’s multiple comparisons tests analysis was used to compare the different groups or Dunette’s multiple comparisons test analysis to compare each experimental group to tumor-free group only. Two-way ANOVA or mixed-effects analysis model and Tukey’s multiple comparisons test were used to compare the tumor growths among the groups overtime. Survival analysis was done using Log-rank (Mantel-Cox) test. Significant p values were annotated as follows *p<0.05, **p<0.01, ***p<0.001, ****p<0.0001.

## Results

### hetIL-15 improves the therapeutic benefit of doxorubicin by reducing metastatic burden in lungs and blood

**Figure 1:**
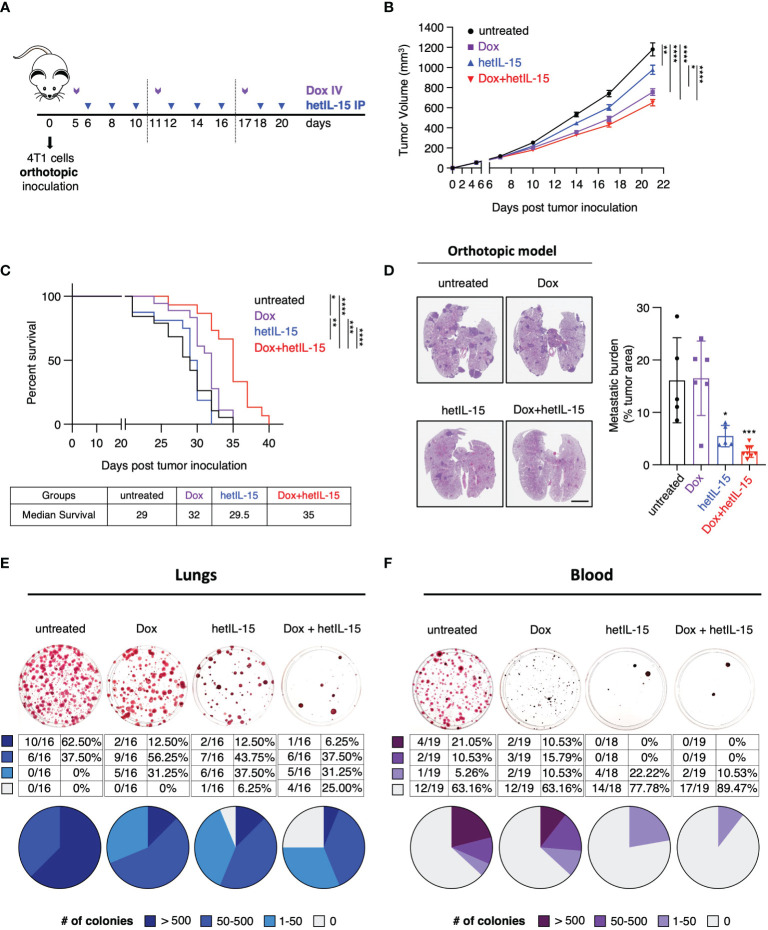
hetIL-15 improves doxorubicin efficacy in extending survival by inhibiting metastatic formation in the lungs and CTCs dissemination in the blood. **(A)** Cartoon showing the treatment design. 0.35-1x10^6^ 4T1 cells were orthotopically inoculated in Balb/c mice and the animals were randomized in four groups: (i) untreated (PBS), (ii) Dox (Doxorubicin)-treated, (iii) hetIL-15-treated and (iv) Dox+hetIL-15-treated. Treatment started on day 5 post tumor inoculation with Dox (5mg/kg IV, purple arrows) once per week, followed by hetIL-15 (3 μg/mouse IP, blue arrows) every other day until day 20. **(B)** Tumor growth curves for each group overtime until day 21. Data were merged from 6 independent studies (n=54-60). Tumor volume (mm^3^) ± SEM for each time point is shown. Statistical analysis was done by mixed effects analysis model and Tukey’s multiple comparisons test. **(C)** Percent survival of the mice in each group. Statistical analysis was performed by Log-rank (Mantel-Cox) test and data merged from two independent experiments (n=15-19). **(D)** Representative images from H&E-stained lung sections (left panel), and the percentage of tumor areas present in each mouse lungs (right panel, asterisks show the significance of difference from the untreated group) presented as mean ± SD for each group. Data derived from one survival study (n=5-8) of **(C)**. Scale bar, 4mm. Statistical analysis was done by one-way ANOVA and Tukey’s multiple comparisons test. **(E, F)** Representative images (upper panels) from clonogenic assays showing SRB-stained tumor colonies derived from lungs **(E)** and blood **(F)**, and tables with the number and the percentage (plotted in pies) of mice with different number of tumor colonies (lower panels) in each group on day 22. Data were merged from 3 independent experiments (n=16) for the lungs and 2 independent experiments (n=18-19) for the blood. Legend shows number of tumor colonies in 4 different ranges. *p < 0.05, **p < 0.01, ***p <0.001, ****p < 0.0001, SD, standard deviation; SEM, standard error of the mean. Image Credit: vector.me.

The anti-cancer activity of hetIL-15, as a single agent or in combination with doxorubicin, was evaluated in the 4T1 murine model of TNBC. 4T1 tumor cells were orthotopically inoculated in Balb/c mice and, when the tumors became palpable, the mice were randomized into four therapeutic groups: untreated (vehicle), doxorubicin, hetIL-15 and combination group. The treatment was performed according to the schedule shown in **Figure 1A**.

The efficacy of the different treatments was initially evaluated by monitoring the growth of the primary tumor and by animal survival. Mice in the three groups that received therapy showed a significant delay in tumor growth compared to the untreated animals (**Figure 1B**) (data pooled from 6 individual studies - **Supplementary Figure S1**). The most prominent effect on tumor delay was observed among mice treated with the combination therapy. However, compared to doxorubicin monotherapy, the two agents together result in a marginal improvement in the control of the primary tumor. When the different treatments were evaluated for animal survival, doxorubicin-treated mice showed a moderate benefit, whereas mice in the combination group had significantly extended survival compared to all the other groups (**Figure 1C**). Although hetIL-15 monotherapy did not show any benefit in survival, when the mice were evaluated for the presence of pulmonary metastatic foci (measured as tumor areas at their individual endpoint), it was found that hetIL-15 exerted a strong anti-metastatic effect (**Figure 1D**). Following the NIH guidelines, animals were euthanized when the primary tumors approached 2cm in one dimension, despite the fact that the mice were not showing any signs of distress or respiratory discomfort. hetIL-15 co-administration with doxorubicin resulted in an even better control of metastasis. The tumor areas covering the lung tissue in untreated and doxorubicin-treated groups were found to be approximately 16%, whereas these areas were reduced to approximately 5.5% and 2.5% in the hetIL-15- and combination-treated mice, respectively (**Figure 1D**).

To evaluate this observation in more depth, the anti-metastatic effect in the lungs was further examined by clonogenic assays. On day 22, whole lung homogenates from each animal were cultured in a selection medium to allow for tumor colony formation. Based on the number of tumor colonies per mouse (represented by each plate), the mice were classified into 4 groups: (i) 0 (no colonies), (ii) 1-50, (iii) 50-500 and (iv) >500 colonies. We found that 62.5% (10/16) of the untreated control mice had more than 500 metastatic colonies, and we observed a decrease in these numbers in the therapeutic groups: 2/16 in the doxorubicin group (12.5%), 2/16 in the hetIL-15 group (12.5%) and 1/16 in the combination group (6.25%) (**Figure 1E**). Importantly, only mice in the hetIL-15 (1 mouse) and combination group (4 mice) were found to have lungs completely free of metastatic cells, revealing the strong anti-metastatic effects of these treatments (**Figure 1E**).

Next, we sought to evaluate the effect of the treatments on CTCs, which are cells capable of initiating metastatic lesions (37). The evaluation was performed by clonogenic assays in blood collected on day 22. The mice were classified into four groups based on the number of tumor colonies, as described above. Of the 19 untreated 4T1 tumor-bearing animals, 7 (36.84%) developed CTC colonies with 4 of these having more than 500 colonies (**Figure 1F**). In contrast, none of the animals in hetIL-15 and combination groups had more than 500 CTC colonies. Interestingly, only 4 out of 18 mice (22.2%) developed CTC colonies in the hetIL-15 group, which however were minimal, as 2 of these animals developed only 15 CTC colonies and the other 2, only 2 colonies. The most prominent effect though was observed in the combination group, where only 2 out of 19 mice (10.53%) were positive for CTC colonies with very low numbers (20 and 1 colonies, respectively). The rest of the mice in both hetIL-15 and combination groups did not develop any colony. Mice in the doxorubicin group also showed a reduction in the number of CTCs, however it was not as effective as in the other treated groups.

Taken together, these data indicate that hetIL-15 shows anti-tumor activity in the 4T1 breast cancer model, mainly by reducing the metastatic burden. hetIL-15 was found to exert its anti-metastatic activity by both decreasing the number of CTCs and by preventing metastasis formation in the lungs. This activity was found to improve the efficacy of doxorubicin, leading ultimately to the extended survival of the combination-treated mice.

### hetIL-15 and combination therapy show local anti-metastatic effect in the lungs

**Figure 2:**
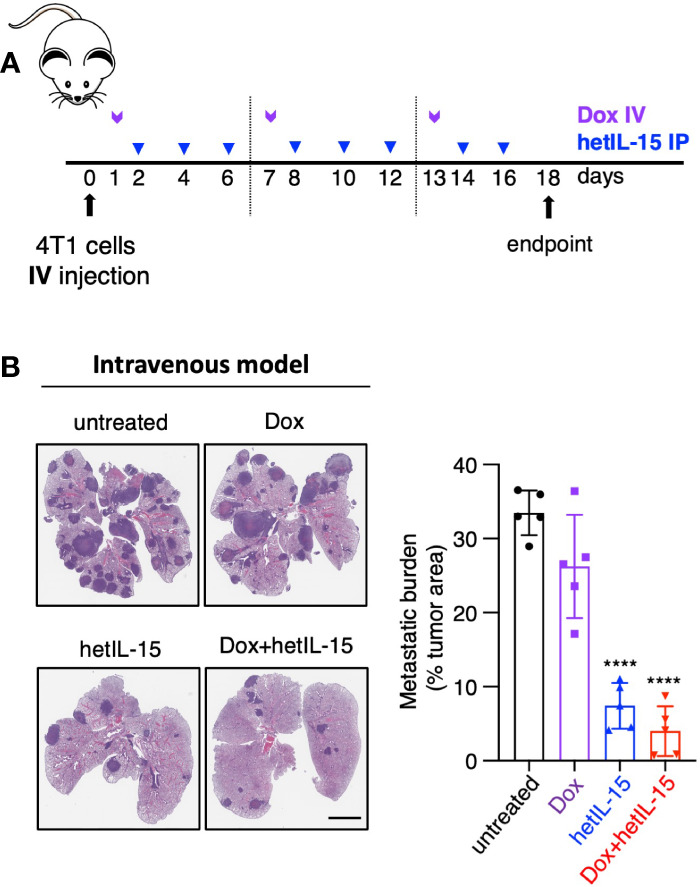
hetIL-15 alone and in combination with doxorubicin exerts local anti-metastatic effect in the lungs. **(A)** Cartoon showing the treatment design. 10^4^ 4T1 cells were injected IV in Balb/c mice and the animals were randomized in four groups: (i) untreated (PBS), (iii) Dox-treated, (iii) hetIL-15-treated and (iv) Dox+hetIL-15-treated. Treatment started one day post tumor cell injection with Dox (5mg/kg IV, purple arrows) once per week, followed by hetIL-15 (3 μg/mouse IP, blue arrows) every other day until day 16. **(B)** Representative images of H&E-stained lung sections (left panel) and the metastatic burden expressed as percentage of tumor area (right panel) in each group on day 18. Percentages presented as mean ± SD for each group. Scale bar, 4mm. Results are from one experiment (n= 5). Statistical analysis was done by one-way ANOVA and Tukey’s multiple comparisons test. Asterisks show the significance of difference from the untreated group, ****p < 0.0001. Image Credit: vector.me.

Given the observation that the treatments decreased the CTCs in the blood, we aimed to study whether the observed anti-metastatic effect in the lungs is a result of fewer cells reaching the tissue. For this purpose, we injected 4T1 tumor cells intravenously into the lateral tail-vein making the lungs the main site of tumor cells seeding. The treatment started 24 hours post cells injection following the schedule shown in **Figure 2A**. On day 18, mice were sacrificed, and their lungs were analyzed by histology for the presence of metastatic foci (expressed as tumor areas). Mice in the untreated and doxorubicin groups showed approximately 30% of the lung area occupied by tumor cells. hetIL-15 treatment decreased the area to 7% and combination therapy to 4% (5-fold and 8-fold decrease, respectively) (**Figure 2B**). Of note, two mice in the combination group had less than 1% of lung surface occupied by tumor. This finding provides evidence that hetIL-15, in addition to CTCs reduction, exerts its anti-metastatic effect by inhibiting the colonization of tumor cells locally in the lung metastatic site. Combination with chemotherapy enhanced this effect.

### hetIL-15 synergizes with doxorubicin to increase CD8^+^T and NK cells and reduce PMN-MDSCs systemically

To identify the mechanisms of the observed anti-cancer effects, we sought to evaluate the immune profile of the 4T1 tumor-bearing mice upon treatment. We have previously reported the role of hetIL-15 on expanding CD8^+^T and NK cells in tumor-bearing mice (19, 20), while others have shown that doxorubicin decreases the myeloid-derived suppressor cells (MDSCs) (38). For these reasons, we analyzed by flow cytometry the blood, spleens, lungs and tumors of tumor-bearing and age-matched tumor-free mice, to monitor changes in the frequencies of CD8^+^T, NK, PMN (polymorphonuclear) - and M (monocytic) -MDSC cells. All tissues were collected two days after the last hetIL-15 dose (day 16).

**Figure 3:**
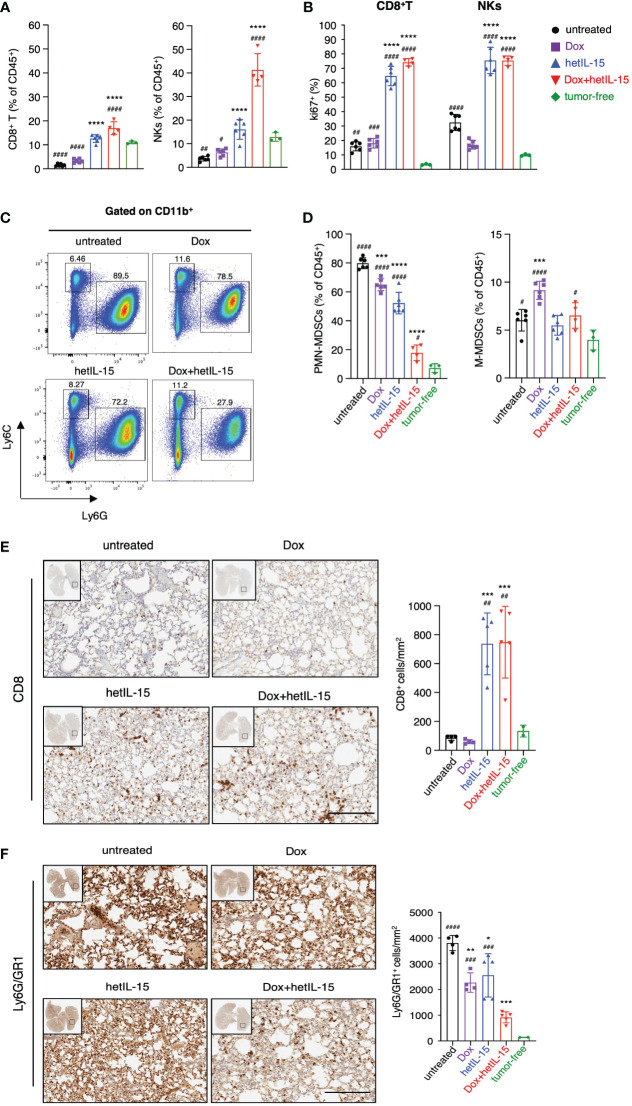
hetIL-15 synergizes with doxorubicin to increase CD8^+^T and NK cells and to reduce PMN-MDSCs in lungs. Mice were treated following the scheme shown in **Figure 1A** and the tissues were harvested on day 16. **(A)** Frequencies and **(B)** Ki67 expression of CD8^+^T and NK cells in the lungs of each group. Data are representative of two independent experiments and bars show the mean ± SD for each group (n= 4-6). **(C)** Dot plots from a representative animal from each group showing the percentages of PMN-MDSCs and M-MDSCs in lungs gated in CD11b^+^. **(D)** PMN-MDSCs and M-MDSCs frequencies in the lungs of each group expressed as percentage of CD45^+^ cells. Data are representative of two independent experiments and bars show the mean ± SD for each group (n= 4-6). **(E, F)** Representative IHC images of lung slides stained with anti-CD8 **(E)** and anti-Ly6G/GR1 **(F)** for each group. The bar graphs show the absolute numbers of CD8^+^ cells/mm^2^ (**E**, right panel) and Ly6G/GR1^+^ cells/mm^2^ (**F**, right panel) of the whole lung area in each group. Scale bar, 200μm. Results were obtained from one experiment; bars show the mean ± SD for each group (n=4-5). Data obtained from lungs of tumor-free mice are also included [n=3 in **(A–D)** and n=2 in **(E, F)**]. Statistical analysis was done by one-way ANOVA and Tukey’s or Dunette’s multiple comparisons test. Asterisks show the significance of difference from the untreated group and hashtags from the tumor-free group, *or ^#^ *p* < 0.05, ** or ^##^ *p* < 0.01, *** or ^###^ *p <*0.001, **** or ^####^ *p* < 0.0001. SD, standard deviation.

Comparison between tumor-bearing mice and tumor-free mice revealed that the disease causes a systemic reduction in the frequencies of both CD8^+^T and NK cells, as revealed by flow cytometry (**Figure 3A**; **Supplementary Figure S2A, D**). This reduction was prevented by hetIL-15 monotherapy, as the mice showed increased CD8^+^T and NK cell frequencies in their lungs (**Figure 3A**), blood and spleen (**Supplementary Figure S2A, D**). Interestingly, co-administration of hetIL-15 with doxorubicin increased both populations to significantly higher levels also in the lungs (**Figure 3A**), blood and spleen (**Supplementary Figure S2A, D**). The increase was statistically significant compared to untreated and both monotherapy groups with p <0.0001 (except the CD8^+^T cells frequencies in the lungs compared to hetIL-15 monotherapy with p=0.0014), which indicated that the two agents synergize to increase CD8^+^T and NK cell frequencies. Both hetIL-15 and combination treatment promoted CD8^+^T and NK cells proliferation (measured by Ki67 expression) in the same tissues (**Figure 3B**; **Supplementary Figure S2B, E**). These data suggest that the observed increased cell frequencies are a result of increased proliferation within the tissues. The lymphocytes phenotype was further evaluated in the lung tissue by measuring the expression of granzyme B, CD69 and bcl-2 (B-cell lymphoma 2) and the immune checkpoint molecules KLRG1 (killer cell lectin-like receptor G1) and PD-1 (Programmed cell death protein 1) (**Supplementary Figure S3**). CD8^+^ T cells from animals treated with hetIL-15 (monotherapy and combination therapy groups) expressed increased levels of CD69 and contained more granzyme B, indicating an activated cytotoxic phenotype. In addition, the cells expressed higher levels of the antiapoptotic molecule bcl-2, suggesting not only enhanced cytotoxicity but also extended survival of these effector cells within the tissue. The expression of these markers on NK cells were particularly increased in mice of the combination group, whereas the expression of granzyme B was also elevated in NKs of the animals treated with hetIL-15 monotherapy. The expression of KLRG1 was also increased in both cell types in animals treated with hetIL-15 (monotherapy and combination therapy groups), and immune checkpoint inhibitor PD-1 was increased in CD8^+^T cells and in animals treated with combination therapy only (PD-1 expression data in NKs are not shown as the percentage of NKs expressing the marker was below 1%). Within tumors, hetIL-15 treatment resulted in enhanced accumulation of NK cells, while the combination therapy increased both CD8^+^T and NK cells, revealing that the treatments trigger the intratumoral infiltration of effector cells (**Supplementary Figure S4A**). The increase of CD8^+^T cells upon combination treatment was significant not only compared to untreated group, but also to both monotherapy groups (p=0.008 and 0.039 to doxorubicin and hetIL-15-monotherapy groups, respectively). In contrast, doxorubicin as monotherapy did not affect either the frequency or the Ki67 expression in these two cell subsets, in any of the tissues (**Figures 3A, B**; **Supplementary Figures S2A-B, D-E, 4A**).

Next, we examined the frequencies of MDSCs as these cells are characterized by the ability to suppress T and NK cell functions (39). Flow cytometry analysis revealed that tumor-bearing animals had increased frequencies of MDSCs compared to tumor-free counterparts. The population of PMN-MDSCs was found to be significantly expanded in the lungs (**Figure 3D**), blood and spleen (**Supplementary Figures S2C, F**), whereas M-MDSCs were found to be increased mostly in the spleen (**Supplementary Figure S2F**). Mice treated with hetIL-15 and combination therapy were found with significantly reduced PMN-MDSCs systemically (**Figures 3C, D**; **Supplementary Figure S2C, F**). Combination therapy showed the most effective reduction, reducing the PMN-MDSCs frequencies close to normal levels, especially in the lungs. This group showed statistically significant decrease of PMN-MDSCs in comparison to the untreated and both monotherapy groups with p<0.0001 (**Figure 3D**). This effect was also prominent in blood (p<0.0001) (**Supplementary Figure S2C**) and spleen (p<0.0001) (**Supplementary Figure S2F**) revealing significant differences in comparison to either monotherapy group. Our results indicate that the two agents have additive effects also in decreasing PMN-MDSCs. Mice treated with doxorubicin monotherapy also had a lower frequency of PMN-MDSCs, although to a lesser extent as compared to the other two treated groups and only in lungs (**Figure 3D**). Interestingly, mice treated with doxorubicin monotherapy showed an increase in the frequency of M-MDSCs (**Figure 3D**; **Supplementary Figures S2C, F**). Finally, mice from all the groups that received therapy showed a reduction in the tumor-infiltrating PMN-MDSC population, which however reached statistical significance only in the mice from the combination group (**Supplementary Figure S4B**). In contrast, the tumor-infiltrating M-MDSC population was increased in all the therapeutic groups (**Supplementary Figure S4B**).

To confirm the findings from the flow cytometry analysis, the absolute number of CD8^+^ and PMN-MDSC cells in the lungs was also evaluated by immunohistochemistry (IHC). Indeed, the numbers of CD8^+^ cells were higher in mice from the hetIL-15 and combination groups (**Figure 3E**), while the number of PMN-MDSC cells was significantly reduced in all the therapeutic groups (**Figure 3F**). These results suggest that the treatments not only affect the relative frequency of these two cell subsets, but also their absolute numbers.

Overall, these data suggest that hetIL-15 and doxorubicin synergize towards an effective antitumor immunity by increasing the CD8^+^T and NK cells and decreasing the PMN-MDSCs in the lungs, blood, and spleen. The intratumoral populations were found to be similarly affected, although to a lesser extent.

### hetIL-15 and combination therapies restore the imbalance of suppressive to effector cell populations in the tumor-bearing mice

To further evaluate the impact of the different treatments on the immune population landscape, we calculated the ratios of MDSCs (both PMN-MDSCs and M-MDSCs) to CD8^+^T and NK cells in blood, spleen, lungs, and tumors from the mice in the therapeutic groups and contrasted the results with data obtained from age-matched tumor-free mice.

**Figure 4:**
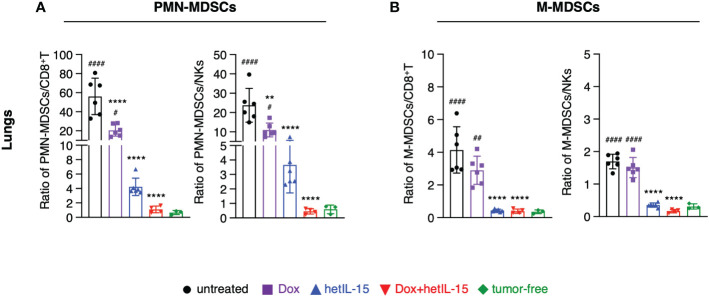
hetIL-15 alone and in combination with doxorubicin decreases the suppressor/effector ratios in the lungs. Ratio of **(A)** PMN-MDSCs to CD8^+^T (left) or NKs (right) and **(B)** M-MDSCs to CD8^+^T (left) or NKs (right) in the lungs of each group. Mice were treated following the treatment schedule shown in **Figure 1A**. The treatment was given for two cycles and the tissues were harvested on day 16. Ratios obtained from tumor-free mice are also included (n=3). Similar results were obtained in two different experiments; bars show the mean ± SD for each group (n=4-6). Statistical analysis was done by one-way ANOVA and Tukey’s or Dunette’s multiple comparisons test. Asterisks show the significance of difference from the untreated group and hashtags from the tumor-free group, ** or ^##^ *p* < 0.01, **** or ^####^ *p* < 0.0001. SD, standard deviation.

All the ratios were found to be significantly increased in the examined tissues of the 4T1 tumor-bearing mice compared to tumor-free animals. Notably, the ratios of PMN-MDSCs to both CD8^+^T and NK cells were more elevated compared to the ratios of M-MDSCs (**Figures 4A, B**; **Supplementary Figures S5A–F**). Combination treatment was the most effective at restoring the ratios back to the levels measured in tumor-free mice, in lungs (**Figure 4A, B**), blood and spleen (**Supplementary Figures S5A–D**). hetIL-15 monotherapy showed a similar trend, although of lower magnitude, in reducing the ratios, but it was as efficient at reducing the M-MDSCs to effector ratios in lungs (**Figure 4B**) and blood (**Supplementary Figure S5B**). Intratumorally, PMN-MDSCs to CD8^+^T ratios were also significantly reduced in the tumor-bearing mice that received the combination therapy while hetIL-15 decreased only the PMN-MDSCs to NKs ratios (**Supplementary Figure S5E**). No significant changes were observed in any treated mice for the M-MDSCs to effector ratios within the tumors (**Supplementary Figure S5F**). Mice treated with doxorubicin alone had also decreased ratios, mostly of PMN-MDSCs to effectors in lungs and blood (**Figure 4A**; **Supplementary Figure S5A**). However, an increase in M-MDSCs to effector ratios was observed as a trend in the spleen (**Supplementary Figure S5D**) and tumors (**Supplementary Figure S5F**) of doxorubicin-treated mice.

Taken together, these data show that hetIL-15 treatment decreases the suppressor to effector cell ratio. This effect is further augmented by the combination with doxorubicin restoring the imbalance induced by the disease and enhancing the antitumor response.

### The pre- and post-surgery administration of hetIL-15 monotherapy or combination therapy eradicate metastatic burden curing the animals

**Figure 5:**
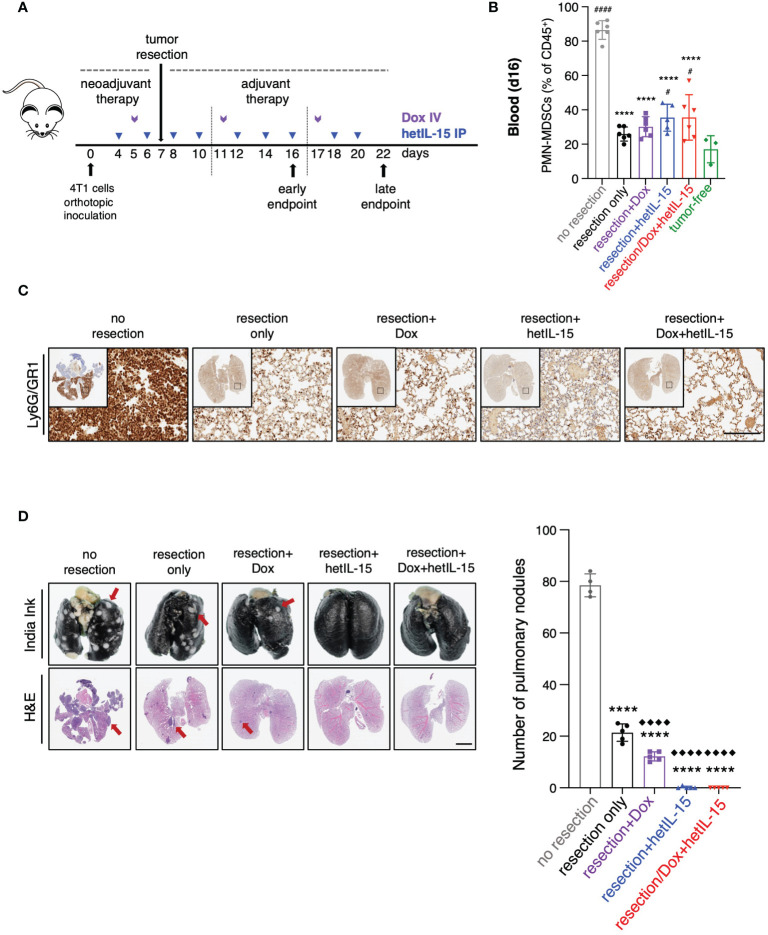
Tumor resection reduces PMN-MDSCs and together with hetIL-15, either alone or in combination with doxorubicin, eradicates metastatic disease. **(A)** Cartoon showing the experimental design with the time of tumor resection and the treatments in neoadjuvant and adjuvant setting. 0.35-1x10^6^ 4T1 cells were orthotopically inoculated in Balb/c mice and tumors were resected one week later. The mice randomized in five groups: (i) no resection, (ii) resection only, (iii) resection+Dox, (iv) resection+hetIL-15 and (v) resection+combinatorial treatment. Treatment started 3 days before surgery with hetIL-15 (3 μg/mouse IP, blue arrows) every other day, followed by doxorubicin (5mg/kg IV, purple arrows) once per week. Treatment endpoints were day 16 (early) or day 22 (late). **(B)** PMN-MDSCs frequencies in blood of each group on day 16. Data obtained from tumor-free mice are also included (n=3). Data are from one experiment; bars shown the mean ± SD for each group (n=5-6). Asterisks show the significance of difference from the non-resected group and hashtags from the tumor-free group ^#^ *p* < 0.05, **** or ^####^ *p* < 0.0001. **(C)** Representative IHC images of Ly6G/GR1-stained lungs from one mouse in each group on day 22. Scale bar, 200μm. Similar results were observed in two independent experiments. **(D)** Representative images from India ink- and H&E-stained lungs (left panels) and number of white tumor nodules (from India ink staining evaluation) in lungs (right panel) are shown as mean ± SD for each group (n=4-5). Results were obtained from two independent experiments. Scale bar, 4mm. Statistical analysis was done by one-way ANOVA and Tukey’s or Dunette’s multiple comparisons test. Asterisks show the significance of difference from the non-resected group and rhombuses from the resection-only group, **** or ^◆◆◆◆^ *p* < 0.0001. SD, standard deviation. Image Credit: vector.me.

It has been previously reported that 4T1 tumor resection reduces MDSCs in the lungs leading to a better control of metastasis (40). Taking this into account and given the observed effects in PMN-MDSCs reduction and metastatic control upon hetIL-15 monotherapy or combination with doxorubicin, we explored the effects of the treatments together with tumor resection. 4T1 tumor cells were orthotopically inoculated in Balb/c mice and the formed tumors were resected one week later. To imitate the neoadjuvant and adjuvant settings (pre- and post-surgery) of the clinical therapeutic schemes (5), the treatments started before surgery and continued afterwards following the administration schedule shown in **Figure 5A**.

The frequency of circulating PMN-MDSCs was analyzed by flow cytometry on day 16 (**Figure 5B**). Mice in the groups that underwent surgery showed approximately 2.5 – 3.5-fold reduction of PMN-MDSCs compared to mice from the unresected group, regardless of the treatment type (**Figure 5B**). Additionally, in all the mice that underwent surgery we found a decrease in the number of Ly6G/GR1 positive cells in their lungs on day 22, indicating a reduction in the absolute numbers of PMN-MDSCs that was again independent of the treatment (**Figure 5C**). These results show that surgery alone results in the depletion of PMN-MDSCs in blood and lungs.

Metastatic disease was evaluated by India Ink staining of the lungs for the detection of pulmonary nodules on day 22 (**Figure 5D**). Primary tumor resection alone was effective in decreasing the number of metastatic nodules in comparison to no resection (mean of 21±3 and 78±4 nodules, respectively) (**Figure 5D**). Remarkably, no metastatic foci were found in the lungs of the mice that underwent surgery and received hetIL-15, either alone or in combination with doxorubicin, revealing that the treatments can eradicate the metastatic disease in the absence of the primary tumor. Mice treated with doxorubicin showed significant foci reduction compared to both non-resected and resected only mice. Similar results were obtained with H&E staining of the lungs (**Figure 5D**).

**Figure 6:**
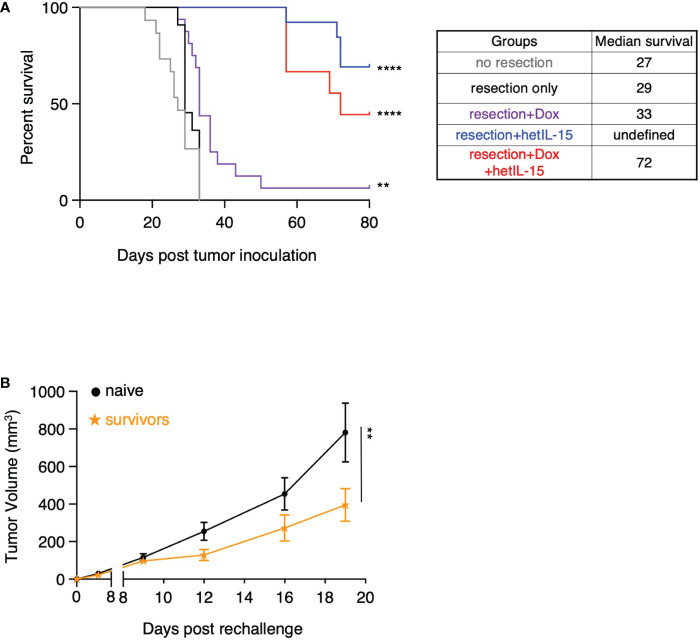
hetIL-15 alone or in combination with doxorubicin cures the resected mice and facilitates the development of tumor-specific immunological memory. **(A)** Survival curves for the animals in each experimental group. Mice were resected and treated following the therapeutic schedule shown in **Figure 5A**. Data merged from two independent studies (n=9-16). Statistical analysis was done by Log-rank (Mantel-Cox) test. **(B)** Tumor growth curves of naive and survivor animals [hetIL-15 and combination group mice merged from (A)] after rechallenge with 5x10^4^ 4T1 cells without any treatment until day 19. Tumor volume (mm^3^) ± SEM for each time point is shown. Statistical analysis was done by two-way ANOVA (n=9-10). **p < 0.01, ****p < 0.0001, SEM, standard error of the mean.

We next evaluated the effects of our therapeutic surgery intervention on the overall survival of the mice (**Figure 6A**). Pre- and post-surgery administration of hetIL-15 monotherapy led to 70% (9 out of 13) cures as the mice did not show any signs of morbidity up to day 80 when the studies were terminated. Similarly, combination treatment led to 45% (4 out of 9) cures of the resected mice, while doxorubicin monotherapy resulted in just 1 cure and marginally extended the median survival to 33 days. None of the non-resected or resected-only mice were cured, and the median survival was similar for both groups (approximately 28 days). Mice that were considered long-term survivors from hetIL-15 (n=7) and combination (n=2) groups were rechallenged with the 4T1 tumor cells. The rechallenge was performed 90 days after the first challenge and the tumor growth was monitored up to day 19 in the absence of any treatment. Survivor animals significantly controlled tumor growth compared to age-matched control animals (challenged for the first time) (**Figure 6B**), indicating the presence of anti-tumor immunity elicited during the first challenge.

Taken together, these data show that pre- and post-surgery administration of hetIL-15 as monotherapy or in combination with doxorubicin can eradicate the metastatic disease which leads to complete cures and allows the development of effective immunological memory against 4T1 tumor cells.

## Discussion

In the present study, we used the immunotherapeutic drug candidate hetIL-15 which has advanced in clinical trials either alone or in combination with the anti-PD-1 antibodies Spartalizumab or Tislelizumab (NCT02452268, NCT04261439). Results from the first-in-human study were recently published reporting that hetIL-15 monotherapy showed stable disease as the best clinical response in 21% of the patients with metastatic or unresectable cancer (22). The data agree with the results obtained in clinical studies using other IL-15 variants (41, 42) and also suggest that combinations should be explored for enhancing IL-15 anti-cancer effects in humans. In this study, we provide supporting evidence that hetIL-15 not only reduces the metastatic disease in the lungs, but also diminishes the number of circulating tumor cells (CTCs). Our findings show the strong anti-metastatic effect of hetIL-15 when administered in combination with doxorubicin and the additive benefits of the two agents in systemically expanding CD8^+^T and NK cells and reducing PMN-MDSCs. Finally, our data also show that tumor resection together with neoadjuvant and adjuvant administration of hetIL-15, either alone or in combination with doxorubicin, results in the cure of approximately half of the treated animals and the development of immunological memory against 4T1 tumor cells.

IL-15 anti-metastatic activity has been reported in preclinical studies, as single agent [rhIL-15 (27, 28), N-803 (23) and RLI (Receptor-Linker-IL-15) (24, 26)] and in combination (N-803 with PD-L1) (30) or fusion with ICIs [N-809 (N-803 fused to two αPD-L1 domains)] (43). In these studies, the use of IL-15 variants as single agents reduced the pulmonary metastatic foci (23, 24, 26–28) and co-administration or fusion with the ICIs increased the anti-metastatic efficacy of IL-15 (30, 43). Here, we show that hetIL-15 monotherapy significantly reduces metastatic disease in the lungs and co-administration with doxorubicin enhances the efficacy. Combination therapy is also more effective in delaying the growth of the primary 4T1 tumor, while hetIL-15 monotherapy shows a moderate effect. Other IL-15 monotherapy variants have not shown any effect on delaying the primary tumor growth (23, 24, 30). This difference could be attributed to the different treatment schemes (routes of delivery and the total amount of the IL-15 given in the different studies). Further improvements that include alternative delivery routes (such as peritumoral administration) or drug delivery systems, could improve the anti-tumor effect (44).

A main finding of the present study is that hetIL-15 diminishes CTCs. CTCs are the precursors of metastatic lesions (37) and there is an increasing interest in their biology and in therapeutic strategies to target them (45). However, CTCs detection is challenging as they are present in low numbers in the blood (46). Here, using clonogenic assays, we detected CTCs in 36.84% of the untreated mice, with more than half of them forming more than 500 CTC colonies. In contrast, the frequency of mice with detectable CTCs was reduced in both hetIL-15- and combination-treated groups, and these mice showed less than 20 CTC colonies. This finding could be the result of several effects targeting different steps of the metastatic process. Reduction of the shedding from the primary site and increased immunosurveillance in the parenchyma or vasculature of the target organs may independently contribute to this protection. It has been suggested that NK cells patrol the circulation in search of malignant cells (47, 48). In this study we show that NKs are increased in animals receiving hetIL-15 or combination treatment. Furthermore, the reduction of PMN-MDSCs upon these treatments may also contribute to the protection as a study has shown that they inhibit NK cytotoxic activity against tumor cells trapped in pulmonary microvessels (49). Additionally, it was recently reported that IL-15 renders NK cells less susceptible to the oxidative stress induced by myeloid cells in the tumor microenvironment (50). Thus, it is possible that hetIL-15 not only expands NKs but also shields them against PMN-MDSCs, helping them to maintain their cytotoxic activity. Further studies are needed to unfold these mechanisms.

Our study suggests that the anti-metastatic effect of hetIL-15 operates not only through diminishing CTCs but also by exerting local effects in the metastatic site, in this case the lungs. This effect could be related to the higher frequencies of activated CD8^+^T and NK cells and the reduced PMN-MDSC frequencies found in the tissue upon hetIL-15 treatment. Increased lung-infiltrating CD8^+^T and NK cells after IL-15 treatment have also been reported in previous studies by our group (15) and others (24). Reduced frequencies of PMN-MDSCs in blood, spleen and lungs could contribute to the anti-metastatic effect as studies have shown that PMN-MDSCs are key regulators of metastasis (51). Others have also reported that MDSC frequencies are reduced upon IL-15/IL-15Ra administration in the Her2/neu+ mammary carcinoma (52). We also show that hetIL-15 decreases the absolute number (not only the frequency) of Ly6G/GR1^+^ cells in the lungs, suggesting that their infiltration is prevented by the cytokine treatment. In agreement with these data, Desbois et al. reported that the CD11b^+^ Ly6G^+^ Ly6Clow cells in the lungs (frequency and absolute counts) were reduced in the 4T1 model upon IL-15-based treatment (24). In line with other studies (30), we did not find major changes in M-MDSCs frequencies, but the M-MDSCs to effectors ratios were restored to normal levels upon hetIL-15 treatment.

Co-administration of hetIL-15 with doxorubicin showed beneficial anti-tumoral effects by increasing the effector populations (CD8^+^T and NK cells) and by reducing the immunosuppressive PMN-MDSCs in all the tissues analyzed. Doxorubicin induces immunogenic cell death facilitating cytotoxic T cells stimulation (53), and also depletes MDSCs (38). This dual function of Doxorubicin helps the immune system to mount effective anti-tumor responses and provides the rationale for combining doxorubicin with IL-15. In our study, the combination of these two agents successfully controlled metastatic disease in the lungs and blood resulting in better animal survival. In earlier studies, IL-15 combined with cyclophosphamide showed better survival and control of metastatic disease in an IV model of rhabdomyosarcoma (31) providing the initial supportive findings of these therapeutic modalities. PMN-MDSCs depletion by doxorubicin may facilitate the expansion of effectors cells mediated by hetIL-15, suggesting a mechanism for the observed enhanced anti-metastatic activity. Although we did not observe a reduction in the frequency of PMN-MDSCs in the blood or spleen of doxorubicin-treated mice, the reduction in the lungs (frequency and absolute numbers) was significant. Moreover, the combination treatment increased the cytotoxicity and proliferation of lymphocytes, which also expressed anti-apoptotic signals. This observation is in line with the strong anti-metastatic effect observed in the lungs of these animals. The lymphocytes also expressed the marker KLRG1 which is expressed in highly differentiated NK and T cells and is related to senescence (54). Nevertheless, there is evidence that KLRG1^+^CD8^+^ T cells are not impaired in effector functions (55), and KLRG1^+^NKs have been shown to protect against lung metastasis (56). Furthermore, the effector cells expressed high levels of the Ki67 marker after combination treatment, indicating that doxorubicin does not negatively affect them even if they actively proliferate upon hetIL-15 administration and also that they are not in exhaustion state. Overall, these findings support the notion of combining doxorubicin, and potentially other anthracyclines, with cytokines like hetIL-15 that stimulate the activation and proliferation of cytotoxic immune cells to improve the treatment outcome.

In an effort to develop an additional clinically relevant approach, we surgically removed the orthotopically implanted 4T1 tumors and treated the animals pre- and post-surgery. Lumpectomy or mastectomy is the standard treatment of TNBC upon clinical detection, and this is followed by chemotherapy. In our experimental model, tumor resection supported by neoadjuvant and adjuvant administration of hetIL-15, either alone or combined with doxorubicin, resulted in the cure of approximately half of the treated mice. The hetIL-15 monotherapy group had a higher percentage of cured mice compared to the combination group, but this difference was not statistically significant. Thus, we do not think that hetIL-15 monotherapy has benefit compared to the combination treatment. Other studies have shown that adjuvant administration of the IL-15 agonist N-803 significantly prolongs animal survival (23, 30). Interestingly, Liu et al. demonstrated that neoadjuvant immunotherapy is superior to adjuvant in controlling metastatic disease in two different murine models of TNBC (including the 4T1 model) (57). We hypothesize that hetIL-15 administration as neoadjuvant (in addition to adjuvant) increases the effector cells and systemically protects the tissues from the cancer cells dissemination associated with surgery. A therapeutic scheme that combines pembrolizumab (anti-PD-1) as neoadjuvant therapy with chemotherapy has been already approved by the FDA for early-stage patients with TNBC (58). Additionally, our data indicate that hetIL-15 treatment facilitates the development of tumor-specific immunological memory. This could represent an effective defense against future relapse caused by dormant cancer cells resistant to chemotherapy (59). Based on the limited number of mice with detectable CTCs when the primary tumor was present, we did not evaluate the CTCs in the resected mice upon the different treatments.

It has been shown that tumor resection leads to decreased metastatic burden in the lungs by depleting MDSCs (40). Although mice in resected-only group had fewer PMN-MDSCs in the periphery and lungs, they did not show benefit in survival compared to the non-resected group. This could be related to the establishment of metastasis prior to tumor resection. In support to this, Bosiljcic et al. (40) reported that although surgery decreases MDSCs, mice are still prone to immunosuppressive functions of PMN-MDSCs for at least 2 weeks after 4T1 tumor resection. In the case of hetIL-15-treated groups, the reduced numbers of PMN-MDSCs in the system of the mice after surgery provided survival benefit, suggesting that hetIL-15 can cure the metastatic disease when the primary tumor is removed and, thus, the tumor-derived factors are heavily decreased. Interestingly, MDSCs have been reported to differentiate into macrophages and mature DCs in the absence of tumor-derived factors (60). The phenotypic analysis of MDSCs upon surgery and treatment would be of interest for future studies.

In summary, our study shows that hetIL-15 exhibits potent anti-metastatic effects in the lungs and reduces the dissemination of tumor cells in peripheral blood, resulting in improved therapeutic benefit in combination with chemotherapy and surgery. Our data suggest that this anti-cancer response is achieved by the expansion of CD8^+^T and NK cells and the reduction of the immunosuppressive PMN-MDSCs caused by hetIL-15 combination with chemotherapy. Importantly, tumor resection accompanied by the pre- and post-surgery administration of hetIL-15 alone or in combination with doxorubicin leads to the cure of the animals and the establishment of immunological memory. Overall, the data presented herein propose that incorporating hetIL-15 in clinical regimens based on doxorubicin and surgery to treat TNBC could control metastasis and thus reduce the disease recurrence rates.

## Data availability statement

The original contributions presented in the study are included in the article/**Supplementary Material**. Further inquiries can be directed to the corresponding authors.

## Ethics statement

The animal study was reviewed and approved by the National Cancer Institute-Frederick Animal Care and Use Committee. NCI-Frederick is accredited by AAALAC International and follows the Public Health Service Policy for the Care and Use of Laboratory Animals. Animal care was provided in accordance with the procedures outlined in the “Guide for Care and Use of Laboratory Animals (National Research Council; 1996; National Academy Press; Washington, D.C.).

## Author contributions

VS, DS, SK, AV and KD performed experiments and analyzed data. BN performed experiments. VS, DS, KD and GP conceived the study and designed the experiments. VS wrote the manuscript. VS, DS, SK, AV, CB, KD and GP reviewed all data and finalized the manuscript. All co-authors reviewed the final manuscript.

## References

[1] Sung H Ferlay J Siegel RL Laversanne M Soerjomataram I Jemal A. Global cancer statistics 2020: GLOBOCAN estimates of incidence and mortality worldwide for 36 cancers in 185 countries. CA: A Cancer J Clin (2021) 71(3):209–49. doi: 10.3322/caac.21660 33538338

[2] Rakha EA Reis-Filho JS Ellis IO. Basal-like breast cancer: A critical review. J Clin Oncol (2008) 26(15):2568–81. doi: 10.1200/JCO.2007.13.1748 18487574

[3] Kumar P Aggarwal R. An overview of triple-negative breast cancer. Arch Gynecol Obstet (2016) 293(2):247–69. doi: 10.1007/s00404-015-3859-y 26341644

[4] Dent R Trudeau M Pritchard KI Hanna WM Kahn HK Sawka CA. Triple-negative breast cancer: clinical features and patterns of recurrence. Clin Cancer Res (2007) 13(15 Pt 1):4429–34. doi: 10.1158/1078-0432.CCR-06-3045 17671126

[5] Bianchini G Balko JM Mayer IA Sanders ME Gianni L. Triple-negative breast cancer: challenges and opportunities of a heterogeneous disease. Nat Rev Clin Oncol (2016) 13(11):674–90. doi: 10.1038/nrclinonc.2016.66 PMC5461122 27184417

[6] Balko JM Giltnane JM Wang K Schwarz LJ Young CD Cook RS. Molecular profiling of the residual disease of triple-negative breast cancers after neoadjuvant chemotherapy identifies actionable therapeutic targets. Cancer Discovery (2014) 4(2):232–45. doi: 10.1158/2159-8290.CD-13-0286 PMC3946308 24356096

[7] Mellman I Coukos G Dranoff G. Cancer immunotherapy comes of age. Nature (2011) 480(7378):480–9. doi: 10.1038/nature10673 PMC3967235 22193102

[8] Cheever MA. Twelve immunotherapy drugs that could cure cancers. Immunol Rev (2008) 222:357–68. doi: 10.1111/j.1600-065X.2008.00604.x 18364014

[9] Carson WE Giri JG Lindemann MJ Linett ML Ahdieh M Paxton R. Interleukin (IL) 15 is a novel cytokine that activates human natural killer cells via components of the IL-2 receptor. J Exp Med (1994) 180(4):1395–403. doi: 10.1084/jem.180.4.1395 PMC2191697 7523571

[10] Zhang X Sun S Hwang I Tough DF Sprent J. Potent and selective stimulation of memory-phenotype CD8+ T cells in vivo by IL-15. Immunity (1998) 8(5):591–9. doi: 10.1016/S1074-7613(00)80564-6 9620680

[11] Klebanoff CA Finkelstein SE Surman DR Lichtman MK Gattinoni L Theoret MR. IL-15 enhances the in vivo antitumor activity of tumor-reactive CD8+ T cells. Proc Natl Acad Sci U.S.A. (2004) 101(7):1969–74. doi: 10.1073/pnas.0307298101 PMC357036 14762166

[12] Munger W Dejoy SQ Jeyaseelan R Torley LW Grabstein KH Eisenmann J. Studies evaluating the antitumor activity and toxicity of interleukin-15, a new T cell growth factor: Comparison with interleukin-2. Cell Immunol (1995) 165(2):289–93. doi: 10.1006/cimm.1995.1216 7553894

[13] Bergamaschi C Stravokefalou V Stellas D Karaliota S Felber BK Pavlakis GN. Heterodimeric IL-15 in cancer immunotherapy. Cancers (Basel) (2021) 13(4):837. doi: 10.3390/cancers13040837 33671252 PMC7922495

[14] Robinson TO Schluns KS. The potential and promise of IL-15 in immuno-oncogenic therapies. Immunol Lett (2017) 190:159–68. doi: 10.1016/j.imlet.2017.08.010 PMC5774016 28823521

[15] Bergamaschi C Rosati M Jalah R Valentin A Kulkarni V Alicea C. Intracellular interaction of interleukin-15 with its receptor alpha during production leads to mutual stabilization and increased bioactivity. J Biol Chem (2008) 283(7):4189–99. doi: 10.1074/jbc.M705725200 18055460

[16] Bergamaschi C Jalah R Kulkarni V Rosati M Zhang GM Alicea C. Secretion and biological activity of short signal peptide IL-15 is chaperoned by IL-15 receptor alpha in vivo. J Immunol (2009) 183(5):3064–72. doi: 10.4049/jimmunol.0900693 PMC7250482 19696432

[17] Chertova E Bergamaschi C Chertov O Sowder R Bear J Roser JD. Characterization and favorable in vivo properties of heterodimeric soluble IL-15.IL-15Ralpha cytokine compared to IL-15 monomer. J Biol Chem (2013) 288(25):18093–103. doi: 10.1074/jbc.M113.461756 PMC3689953 23649624

[18] Bergamaschi C Bear J Rosati M Beach RK Alicea C Sowder R. Circulating IL-15 exists as heterodimeric complex with soluble IL-15Ralpha in human and mouse serum. Blood (2012) 120(1):e1–8. doi: 10.1182/blood-2011-10-384362 PMC3390963 22496150

[19] Ng SSM Nagy BA Jensen SM Hu X Alicea C Fox BA. Heterodimeric IL15 treatment enhances tumor infiltration, persistence, and effector functions of adoptively transferred tumor-specific T cells in the absence of lymphodepletion. Clin Cancer Res (2017) 23(11):2817–30. doi: 10.1158/1078-0432.CCR-16-1808 PMC5805388 27986749

[20] Bergamaschi C Pandit H Nagy BA Stellas D Jensen SM Bear J. Heterodimeric IL-15 delays tumor growth and promotes intratumoral CTL and dendritic cell accumulation by a cytokine network involving XCL1, IFN-γ, CXCL9 and CXCL10. J Immunother Cancer (2020) 8(1). doi: 10.1136/jitc-2020-000599 PMC7254133 32461349

[21] Stellas D Karaliota S Stravokefalou V Nagy BA Felber BK Pavlakis GN. Abstract 3259: heterodimeric IL-15 monotherapy results in complete regression of EO771 murine breast tumors through cDC1-lymphocyte interactions and induction of antitumor immunity. Cancer Res (2019) 79(13_Supplement):3259–9. doi: 10.1158/1538-7445.AM2019-3259

[22] Conlon K Watson DC Waldmann TA Valentin A Bergamaschi C Felber BK. Phase I study of single agent NIZ985, a recombinant heterodimeric IL-15 agonist, in adult patients with metastatic or unresectable solid tumors. J ImmunoTher Cancer (2021) 9(11):e003388. doi: 10.1136/jitc-2021-003388 34799399 PMC8606766

[23] Kim PS Kwilas AR Xu W Alter S Jeng EK Wong HC. IL-15 superagonist/IL-15RαSushi-Fc fusion complex (IL-15SA/IL-15RαSu-Fc; ALT-803) markedly enhances specific subpopulations of NK and memory CD8+ T cells, and mediates potent anti-tumor activity against murine breast and colon carcinomas. Oncotarget (2016) 7(13):16130–45. doi: 10.18632/oncotarget.7470 PMC4941302 26910920

[24] Desbois M Béal C Charrier M Besse B Meurice G Cagnard N. IL-15 superagonist RLI has potent immunostimulatory properties on NK cells: implications for antimetastatic treatment. J ImmunoTher Cancer (2020) 8(1):e000632. doi: 10.1136/jitc-2020-000632 32532840 PMC7295443

[25] Kobayashi H Dubois S Sato N Sabzevari H Sakai Y Waldmann TA. Role of trans-cellular IL-15 presentation in the activation of NK cell-mediated killing, which leads to enhanced tumor immunosurveillance. Blood (2005) 105(2):721–7. doi: 10.1182/blood-2003-12-4187 15367431

[26] Bessard A Solé V Bouchaud G Quéméner A Jacques Y. High antitumor activity of RLI, an interleukin-15 (IL-15)–IL-15 receptor α fusion protein, in metastatic melanoma and colorectal cancer. Mol Cancer Ther (2009) 8(9):2736–45. doi: 10.1158/1535-7163.MCT-09-0275 19723883

[27] Yu P Steel JC Zhang M Morris JC Waldmann TA. Simultaneous blockade of multiple immune system inhibitory checkpoints enhances antitumor activity mediated by interleukin-15 in a murine metastatic colon carcinoma model. Clin Cancer Res (2010) 16(24):6019–28. doi: 10.1158/1078-0432.CCR-10-1966 PMC3005104 20924130

[28] Tang F Zhao L Jiang Y Ba D Cui L He W. Activity of recombinant human interleukin-15 against tumor recurrence and metastasis in mice. Cell Mol Immunol (2008) 5(3):189–96. doi: 10.1038/cmi.2008.23 PMC4651289 18582400

[29] Gillgrass A Gill N Babian A Ashkar AA. The absence or overexpression of IL-15 drastically alters breast cancer metastasis via effects on NK cells, CD4 T cells, and macrophages. J Immunol (2014) 193(12):6184–91. doi: 10.4049/jimmunol.1303175 25355926

[30] Knudson KM Hicks KC Alter S Schlom J Gameiro SR. Mechanisms involved in IL-15 superagonist enhancement of anti-PD-L1 therapy. J Immunother Cancer (2019) 7(1):82. doi: 10.1186/s40425-019-0551-y 30898149 PMC6429734

[31] Chapoval AI Fuller JA Kremlev SG Kamdar SJ Evans R. Combination chemotherapy and IL-15 administration induce permanent tumor regression in a mouse lung tumor model: NK and T cell-mediated effects antagonized by b cells. J Immunol (1998) 161(12):6977–84. 9862733

[32] Chen G Emens LA. Chemoimmunotherapy: reengineering tumor immunity. Cancer Immunol Immunother (2013) 62(2):203–16. doi: 10.1007/s00262-012-1388-0 PMC3608094 23389507

[33] von Minckwitz G Martin M. Neoadjuvant treatments for triple-negative breast cancer (TNBC). Ann Oncol (2012) 23:vi35–9. doi: 10.1093/annonc/mds193 23012300

[34] Kleinman HK Martin GR. Matrigel: basement membrane matrix with biological activity. Semin Cancer Biol (2005) 15(5):378–86. doi: 10.1016/j.semcancer.2005.05.004 15975825

[35] Pulaski BA Ostrand-Rosenberg S. Mouse 4T1 breast tumor model. Curr Protoc Immunol (2001). doi: 10.1002/0471142735.im2002s39 18432775

[36] Orellana EA Kasinski AL. Sulforhodamine b (SRB) assay in cell culture to investigate cell proliferation. Bio-protocol (2016) 6(21). doi: 10.21769/BioProtoc.1984 PMC5448418 28573164

[37] Baccelli I Schneeweiss A Riethdorf S Stenzinger A Schillert A Vogel V. Identification of a population of blood circulating tumor cells from breast cancer patients that initiates metastasis in a xenograft assay. Nat Biotechnol (2013) 31(6):539–44. doi: 10.1038/nbt.2576 23609047

[38] Alizadeh D Trad M Hanke NT Larmonier CB Janikashvili N Bonnotte B. Doxorubicin eliminates myeloid-derived suppressor cells and enhances the efficacy of adoptive T-cell transfer in breast cancer. Cancer Res (2014) 74(1):104–18. doi: 10.1158/0008-5472.CAN-13-1545 PMC3896092 24197130

[39] Veglia F Sanseviero E Gabrilovich DI. Myeloid-derived suppressor cells in the era of increasing myeloid cell diversity. Nat Rev Immunol (2021) 21(8):485–98. doi: 10.1038/s41577-020-00490-y PMC7849958 33526920

[40] Bosiljcic M Cederberg RA Hamilton MJ LePard NE Harbourne BT Collier JL. Targeting myeloid-derived suppressor cells in combination with primary mammary tumor resection reduces metastatic growth in the lungs. Breast Cancer Res (2019) 21(1):103. doi: 10.1186/s13058-019-1189-x 31488209 PMC6727565

[41] Miller JS Morishima C McNeel DG Patel MR Kohrt HEK Thompson JA. A first-in-Human phase I study of subcutaneous outpatient recombinant human IL15 (rhIL15) in adults with advanced solid tumors. Clin Cancer Res (2018) 24(7):1525–35. doi: 10.1158/1078-0432.CCR-17-2451 PMC6741437 29203590

[42] Margolin K Morishima C Velcheti V Miller JS Lee SM Silk AW. Phase I trial of ALT-803, a novel recombinant IL15 complex, in patients with advanced solid tumors. Clin Cancer Res (2018) 24(22):5552–61. doi: 10.1158/1078-0432.CCR-18-0945 PMC6239933 30045932

[43] Knudson KM Hicks KC Ozawa Y Schlom J Gameiro SR. Functional and mechanistic advantage of the use of a bifunctional anti-PD-L1/IL-15 superagonist. J ImmunoTher Cancer (2020) 8(1):e000493. doi: 10.1136/jitc-2019-000493 32303618 PMC7204804

[44] Stravokefalou V Stellas D Karaliota S Nagy B Guerin T Kozlov S. Abstract 2727: Heterodimeric IL-15 (hetIL-15) affects conventional dendritic cells and a distinct novel dendritic cell population in different mouse cancer models of breast and pancreatic cancer. Cancer Res (2021) 81(13_Supplement):2727–7. doi: 10.1158/1538-7445.AM2021-2727

[45] Zhong X Zhang H Zhu Y Liang Y Yuan Z Li J. Circulating tumor cells in cancer patients: developments and clinical applications for immunotherapy. Mol Cancer (2020) 19(1):15. doi: 10.1186/s12943-020-1141-9 31980023 PMC6982393

[46] Hattori M Nakanishi H Yoshimura M Iwase M Yoshimura A Adachi Y. Circulating tumor cells detection in tumor draining vein of breast cancer patients. Sci Rep (2019) 9(1):18195. doi: 10.1038/s41598-019-54839-y 31796846 PMC6890763

[47] Barlozzari T Reynolds CW Herberman RB. In vivo role of natural killer cells: involvement of large granular lymphocytes in the clearance of tumor cells in anti-asialo GM1-treated rats. J Immunol (1983) 131(2):1024–7. 6863925

[48] Lambert AW Pattabiraman DR Weinberg RA. Emerging biological principles of metastasis. Cell (2017) 168(4):670–91. doi: 10.1016/j.cell.2016.11.037 PMC5308465 28187288

[49] Spiegel A Brooks MW Houshyar S Reinhardt F Ardolino M Fessler E. Neutrophils suppress intraluminal NK cell-mediated tumor cell clearance and enhance extravasation of disseminated carcinoma cells. Cancer Discov (2016) 6(6):630–49. doi: 10.1158/2159-8290.CD-15-1157 PMC4918202 27072748

[50] Yang Y Neo SY Chen Z Cui W Chen Y Guo M. Thioredoxin activity confers resistance against oxidative stress in tumor-infiltrating NK cells. J Clin Invest (2020) 130(10):5508–22. doi: 10.1172/JCI137585 PMC7524507 32673292

[51] Ouzounova M Lee E Piranlioglu R El Andaloussi A Kolhe R Demirci MF. Monocytic and granulocytic myeloid derived suppressor cells differentially regulate spatiotemporal tumour plasticity during metastatic cascade. Nat Commun (2017) 8:14979. doi: 10.1038/ncomms14979 28382931 PMC5384228

[52] Guo S Smeltz RB Nanajian A Heller R. IL-15/IL-15Rα heterodimeric complex as cancer immunotherapy in murine breast cancer models. Front Immunol (2021) 11(3712). doi: 10.3389/fimmu.2020.614667 PMC7897681 33628206

[53] Casares N Pequignot MO Tesniere A Ghiringhelli F Roux S Chaput N. Caspase-dependent immunogenicity of doxorubicin-induced tumor cell death. J Exp Med (2005) 202(12):1691–701. doi: 10.1084/jem.20050915 PMC2212968 16365148

[54] Borys SM Bag AK Brossay L Adeegbe DO. The yin and yang of targeting KLRG1(+) tregs and effector cells. Front Immunol (2022) 13:894508. doi: 10.3389/fimmu.2022.894508 35572605 PMC9098823

[55] Sarkar S Kalia V Haining WN Konieczny BT Subramaniam S Ahmed R. Functional and genomic profiling of effector CD8 T cell subsets with distinct memory fates. J Exp Med (2008) 205(3):625–40. doi: 10.1084/jem.20071641 PMC2275385 18316415

[56] Malaisé M Rovira J Renner P Eggenhofer E Sabet-Baktach M Lantow M. KLRG1+ NK cells protect T-bet-deficient mice from pulmonary metastatic colorectal carcinoma. J Immunol (2014) 192(4):1954–61. doi: 10.4049/jimmunol.1300876 24415778

[57] Liu J Blake SJ Yong MC Harjunpää H Ngiow SF Takeda K. Improved efficacy of neoadjuvant compared to adjuvant immunotherapy to eradicate metastatic disease. Cancer Discov (2016) 6(12):1382–99. doi: 10.1158/2159-8290.CD-16-0577 27663893

[58] Schmid P Cortes J Pusztai L McArthur H Kümmel S Bergh J. Pembrolizumab for early triple-negative breast cancer. New Engl J Med (2020) 382(9):810–21. doi: 10.1056/NEJMoa1910549 32101663

[59] Sosa MS Bragado P Aguirre-Ghiso JA. Mechanisms of disseminated cancer cell dormancy: an awakening field. Nat Rev Cancer (2014) 14(9):611–22. doi: 10.1038/nrc3793 PMC4230700 25118602

[60] Narita Y Wakita D Ohkur T Chamoto K Nishimura T. Potential differentiation of tumor bearing mouse CD11b+Gr-1+ immature myeloid cells into both suppressor macrophages and immunostimulatory dendritic cells. BioMed Res (2009) 30(1):7–15. doi: 10.2220/biomedres.30.7 19265258

