# Supplementary material for: Heterodimeric IL-15 (hetIL-15) reduces circulating tumor cells and metastasis formation improving chemotherapy and surgery in 4T1 mouse model of TNBC
Source: Front Immunol. 2023 Jan 13;13:1014802. doi: 10.3389/fimmu.2022.1014802 (PMC9880212; doi:10.3389/fimmu.2022.1014802)
Supplement: Supplementary file 1 [file DataSheet_1.docx]

Supplementary Material

**Supplementary Figure S1.** Tumor growth curves for each group of six individual studies pooled in Figure 1B (total n=54-60). Tumor volume (mm^3^)±SEM for each time point is shown up to day 21. Statistical analysis was done by mixed effects analysis model and Tukey’s multiple comparisons test. **p* < 0.05, ***p* < 0.01, ****p* <0.001, *****p* < 0.0001, SEM, standard error of the mean.

**Supplementary Figure S2.** hetIL-15 synergizes with doxorubicin to increase the frequencies and the proliferation of CD8^+^T and NK cells and to reduce the frequencies of PMN-MDSCs in blood and spleen. 4T1 tumor-bearing mice were treated following the schedule shown in Figure 1A and the tissues were harvested on day 16. Frequencies and Ki67 expression of CD8^+^T and NK cells, and frequencies of PMN-MDSCs and M-MDSCs in blood (A-C) and spleen (D-F) for each group. Data obtained from tumor-free mice are also included (n=3). Data are representative of two independent experiments; bars show the mean±SD for each group (n= 4-6). Statistical analysis was done by one-way ANOVA and Tukey’s or Dunette’s multiple comparisons test. Asterisks show the significance of difference from the untreated group and hashtags from the tumor-free group, *or # p < 0.05, ** or ## p < 0.01, *** or ### p <0.001, **** or #### p < 0.0001. SD, standard deviation.

**Supplementary Figure S3.** Both activation and inhibitory markers are elevated in CD8^+^ T and NK cells upon combination of hetIL-15 with doxorubicin. 4T1 tumor-bearing mice were treated following the schedule shown in Figure 1A and the tissues were harvested on day 16. Granzyme B, CD69, bcl-2, KLRG1 and PD-1 expression of CD8+T (upper panels) and of NK cells (lower panels) for each group. Data obtained from tumor-free mice are also included (n=2). Data are obtained from one experiment; bars show the mean±SD for each group (n= 3-7). Statistical analysis was done by one-way ANOVA and Tukey’s or Dunette’s multiple comparisons test. Asterisks show the significance of difference from the untreated group and hashtags from the tumor-free group, *or # p < 0.05, ** or ## p < 0.01, *** or ### p <0.001, **** or #### p < 0.0001. SD, standard deviation.

**Supplementary Figure S4**. hetIL-15 in combination with doxorubicin increases the frequencies of CD8+T and NK cells and reduces the frequencies of PMN-MDSCs in tumor. Mice were treated according to the treatment schedule shown in Figure 1A and the tumors were harvested on day 16. Frequencies of (A) CD8+T and NK cells, and (B) PMN-MDSCs and M-MDSCs, in tumors of each group. Data are merged from two experiments; bars show the mean±SD for each group (n= 5-9). Statistical analysis was done by one-way ANOVA and Tukey’s multiple comparisons test. Asterisks show the significance of difference from the untreated group, *p < 0.05, **p < 0.01, ***p <0.001, ****p < 0.0001. SD, standard deviation

**Supplementary** **Figure S5**. hetIL-15 alone or in combination with doxorubicin decreases the suppressor/effector ratios in blood and spleens and partially in tumors. Ratio of PMN-MDSCs to CD8^+^T (left) or NKs (right) and M-MDSCs to CD8^+^T (left) or NKs (right) in blood (A-B), spleen (C-D) and tumors (E-F) in each group. Mice were treated following the schedule shown in Figure 1A and the tissues were harvested on day 16. Ratios obtained from blood and spleen of tumor-free mice are also included (n=3). Similar results were obtained in two different experiments from blood and spleen (n=4-6). Data from tumors merged from two independent experiments (n=5-9). Bars show the mean±SD for each group. Statistical analysis was done by one-way ANOVA and Tukey’s or Dunette’s multiple comparisons test. Asterisks show the significance of difference from the untreated group and hashtags from the tumor-free group, *or ^#^ *p* < 0.05, ** or ^##^ *p* < 0.01, *** or ^###^ *p* <0.001, **** or ^####^ *p* < 0.0001. ns, non-significant; SD, standard deviation.
